# The effects of a non-school setting on quantity and quality of biology knowledge of primary students–A pilot study

**DOI:** 10.1371/journal.pone.0284300

**Published:** 2023-04-27

**Authors:** Ludmila Malariková, Kristýna Machová, Ivona Svobodová, Radka Prochazková, Aneta Makovcová

**Affiliations:** 1 Department of Ethology and Companion Animal Science, Faculty of Agrobiology, Food, and Natural Resources, Czech University of Life Sciences Prague, Prague, Czech Republic; 2 The Department of Statistics, Faculty of Economics and Management, Czech University of Life Sciences Prague, Prague, Czech Republic; Tallinn University: Tallinna Ulikool, ESTONIA

## Abstract

To increase the effectiveness of education is appropriate to incorporate varied teaching methods with multisensory stimulation and with an emphasis on personal and emotional experiences. This study aims to compare the knowledge of biology subject matter acquired by second and fourth-grade primary students. The lesson occurred at a farm in the experimental group and at school in the control group. Students’ knowledge levels were assessed before the lesson, after the lesson, after 14 days, after a month, and after six months. When the levels of knowledge after the lesson were compared between the groups, significantly better results (p = 0.001) were recorded in students in the control group. Another 14 days after the lesson, there was no significant difference in knowledge between the groups (p = 0.848). The same results were obtained after a month (p = 0.760) and after six months (p = 0.649). In the experimental group, the intra-group analysis did not show any significant difference in the levels of knowledge before and after the lesson; it was recorded only after 14 days. In contrast, the control group showed a significant improvement in knowledge right after the lesson, which was not observed later on. Most often, this phenomenon was observed in second-grade students. The presence of animals in an educational setting can add many benefits, such as mental well-being, an increase in empathy, or support for socio-emotional development. Since the levels of subject matter knowledge acquired at a farm and at school were similar, it seems that farm lessons should not negatively impact education, and it offers many related positive effects.

## Introduction

Effective teaching combines different teaching methods and activities. Some contemporary articles describe an effort to enrich teaching with multimedia technologies and thus make the subject more attractive for children [1]. The modern trend is mainly the virtualization of the teaching process, related to integrating multimedia approaches into traditional teaching. On the one hand, these technologies, mainly using the visual sensory channel, contribute to the reduction of monologic teaching methods; on the other hand, there is still no transfer from a theoretical (virtual) to a practical (realistic) explanation [2]. Multimedia teaching involving technology seems to be more effective than mere monologic methods. The problem is that with the development of technologies that have become part of our daily lives, people began to spend more time at home in front of televisions, mobile phones, or computers [3]. Currently, the age at which children start working with tablets or mobile phones is rapidly decreasing [4] and this has a negative effect on children’s patterns of physical activity [5] and socio-emotional development including their ability to empathize and establish social contacts [6]. Therefore, it seems appropriate to look for other possibilities to enrich traditional teaching.

The problem of traditional school teaching is that often is impoverished in sensory stimuli [2]. In order to maximize the effectiveness of children’s learning, it is necessary to ensure that education is sufficiently multifarious [7]. It seems appropriate to involve as many senses as possible in acquiring information. One of the essential options is to involve the sense of touch and to allow students to feel the objects of learning, thus taking them from the theoretical to the realistic sphere. Verbal monologic teaching methods prevail in formal education, relying on the use of spoken explanations only, which, moreover, are not very practical from this point of view since most people belong to the so-called visual types, which preferably receive information by sight [8].

However, when multisensory teaching is combined with an emphasis on personal experience, students remember the topic discussed even longer and, in more detail, compared to learning involving only one or two senses [2, 9]. Integrating living entities that possess unique personalities and characteristics changes the traditional classroom environment, and consequently can change students’ responses to learning. Many nature-oriented programs rely on the combination of personal experience, a possible real relationship created with the subject of teaching, and the opportunity to physically get to know the studied topic in its natural environment [10–12]. These could also have a positive effect on the level of information acquisition. The absorption and duration of retention of this experienced information could then be longer than during traditional teaching [13]. And precisely these topics, in the case of teaching biology, can be supported by teaching on a farm with the participation of the animals being discussed in their natural environment.

The inclusion of animals in teaching is called animal-assisted education (AAE). AAE is relatively well described in relation to its involvement in teaching reading skills to children [14]. A meta-analysis focusing on the effects of dog-assisted interventions on learning showed that the inclusion of animals in education can positively impact learning outcomes in the social, emotional, and behavioral fields or even the reading skills of pupils. Of those three, it was the area of social outcomes, which was affected the most, whereas reading was influenced the least [15]. Another literature review similarly noted an increased motivation to read, more confidence and lower anxiety in participants of dog reading programs for children with special educational needs, although lacking any evidence of improvement of reading skills themselves [14]. In the area of cognitive abilities, studies included in the review were scarce and presented mixed results, such as no influence on intellectual quotient (IQ), but on the other hand a positive impact on memory and in some cases even categorization tasks, especially when it came to animated objects [14].

There are also studies describing the involvement of animals in teaching that takes place directly in classrooms [15]. However, there is very little literature focused directly on the involvement of animals in teaching outside the school environment, specifically on the farm. Even though a recreational and an educational function of farms is lately increasing in Europe [16] so far, the research on farm-based intervention concentrated more on psycho-social outcomes rather than academic performance [12, 17, 18]. The presence of animals, nature, and the farm itself with its simple schedule and rules help create an atmosphere of acceptance, thus a safe environment for learning, which easily captures attention and serves as a strong motivation to participate [12]. Farm-based interventions often aim at people with behavioral and emotional issues [17] or even a psychiatric diagnosis [19]. Most of the current educational farm programs are all concentrate on teaching healthy habits, work ethic, practical skills, or psycho-social development rather than improving the process of acquiring academic knowledge [10, 16, 17].

As mentioned even less literature is focused on the evaluation of the impact on the academic skills of the students involved. Still, one recent study compared how the environment of a farm influenced the learning process in terms of the pupils’ perceived experience as well as academic performance in a test, compared to traditional classroom learning. At a farm, the students got significantly better test scores, especially when it came to farm-related topics, which was even more apparent when long-term knowledge retention was tested. Interviews showed that the pupils themselves perceived the learning process as easier and more effective. Pupils were able to build a relationship with the topic which made learning more personal and meaningful [18].

These findings suggest that farm-based animal assisted education could enrich the learning process by introducing certain benefits, since memorization of the presented subject matter is influenced by individual experience [20]. Personal experiences and exciting events are stored in a more durable and persistent episodic memory than semantic memory [9]. The stored memory trace is thus more complex and forms a more significant number of associative connections facilitating its later recall [21]. Teaching should be linked to certain physical activities that are episodic in nature, i.e., for example, dramatization, staged teaching methods, work activities, didactic play, etc. However, it is also true that each of us experiences any given situation differently, in our way, based on individually developed personality components and previous experiences, needs, and preferences [9].

Including live animals in the teaching process combines all of the abovementioned aspects. It can make the lessons exciting and enriched with new stimuli, as recommended by [2, 9]. Moreover, it is associated with individual experience [20] influenced by the child’s relationship with domestic and farm animals. AAE tends to be interactive and offers a range of emotional experiences [22] that play a crucial role in attaining knowledge [23]. If children can walk up to animals and touch them, they can feel their body shape and surface texture in a truly authentic way, which is an entirely different perception than if they read about the subject matter in books. Lessons conceived as animal-assisted education offer children real, authentic experiences [22]. Therefore, a possible way to promote motivation and learning is to integrate live animals into biology lessons [24]. So far, this topic has only been described very marginally, and there are currently very few studies describing the effect of on-farm teaching involvement [12, 25] on the extent and length of retention of acquired information [13].

This study aims to map the effect of engaging in animal-assisted education at a farm on the quantity of information the student took away from the lesson compared to traditional classroom-based learning. Another objective is to evaluate if a non-school education setting will impact the retention of the information learned even two weeks, one month, and six months later.

## Materials and methods

### Participants

A total of 98 students participated in the study (60 students were boys and 38 were girls). Forty-one students in this study attended the second grade, and 57 students attended the fourth grade. In the experimental group, the ages of the participants (n = 50; 27 men, 23 women) who completed the course of the study ranged from 7 to 10 years (mean = 8.98, median = 10, standard deviation 1.15). The experimental group included 22 second-grade students and 28 students attending fourth grade. In the control group, the participants (n = 48; 37 men, 11 women) were aged 7 to 10 years (mean = 9.08, median = 10, standard deviation = 1.05). There were 23 second-grade students and 30 fourth-grade students in this group. All participants attended a 60-minute lesson, which took place at a farm in the experimental group or a standard classroom setting in the control group. The length of both lessons was the same. The second class and the fourth class attended the lesson on a different day.

As the study was conducted during the Covid-19 pandemic, the study was burdened with higher morbidity or quarantine of the students due to the disease. Ninety-eight respondents completed the test to assess student’s knowledge at the beginning of the study, and 93 students after the end. After 14 days, the questionnaire was completed by 59 students, after one month by 79 students, and after six months by 76 students.

The experimental group was given a lecture according to the topic of that particular lesson, which was based on the school curriculum and supplemented by the presence of children on a farm, where they had the opportunity to see the environment where farm animals lived and had a chance to be in physical contact with the animals. The control group received a classical lesson, which consisted of identical subject matter and included the use of various paper materials in the form of work papers and pictures.

The children’s parents agreed to involve their children in animal-assisted education at a farm as it is a standard part of their school’s curriculum. The testing procedures described here were conducted following the ethical standards of the Ethics Committee of Lincoln University in the UK and the Declaration of Helsinki as amended. The study was approved by the Institutional Review Board of the Czech University of Life Sciences in Prague, and all experiments were conducted following relevant guidelines and regulations.

### General procedures

The lessons were taught to second and fourth-grade primary school students in the Czech Republic, and the children learned about cows, goats, rabbits, and chickens. The teaching was tailored for the second and fourth-grade students according to their current study plan. Before the lesson at school or the lecture at the farm started, the pupils wrote a test to map the level of knowledge with which they came in. The students then completed the same test after the lesson or lecture and then again 14 days, one month, and six months later.

The test for the second and fourth grades had the same number of questions and the same number of possible points, but its content was adapted to the details taught in the given grade according to the pedagogical plan. The test for the second grade included questions on baby animals, the valuable products that can be obtained from livestock, or questions about what the animals eat. The last task was to describe the body of a cow. The test for fourth-grade pupils assessed the same parameters but in more detail. Thus, in the area of baby animals, students were asked about the number of animals in a litter or their hair coat, or they had to list the names of the offspring of each species on their own. For this study, the percentage correctness of the whole test was evaluated.

The farm program took place within a short distance from the school (3 km), so the students walked to the farm on foot. Arriving at the farm, there was a rest period of about half an hour, during which the lecturer introduced herself to the children and told them what would happen and how to behave in the presence of the animals since the pupils do not usually visit the farm. Then the children took the first test to assess their initial knowledge. After taking the test, the pupils went to the animals, where they were allowed to move around and get familiar with them for 15 minutes to get used to their presence before the lesson started. After acclimatization, the actual teaching of the curriculum began, which lasted 60 minutes and covered first goats and then cows. In their studies, Hawkins et al. [26] (11) and Burich & Williams [27] (12, 13) point out that children have inadequate knowledge regarding the needs of livestock; Hawkins et al. [26] (11) report that children lack knowledge regarding species-specific needs of animals.

On the other hand, they are not indifferent to farm animal welfare. They are just unaware of it. Children, who wanted to, could go among the animals and pet the calf for a while. Afterward, the teaching covered chickens and rabbits. The same procedure was followed with the pupils taught in the classroom. Again, the explanation lasted 60 minutes, but there was no acclimatization phase before the explanation. The teacher only introduced herself and got acquainted with the class. In both cases, the lecturer was the same person, and the class teacher was present throughout the lesson.

### Data analysis

In addition to descriptive statistics, selected methods of statistical induction were used to analyze the primary data. Before the statistical analysis, an exploratory data analysis was carried out to verify the assumptions for the subsequent statistical processing (these are, in particular, the independence or dependence of the samples, homogeneity, and normality of the distribution [28]. The exact Shapiro-Wilk normality test tested whether the values sufficiently fulfilled this condition. Since the data did not meet the normality of distribution and homoscedasticity (conformity of variances) concerning the sample size and the presence of outliers and extreme values, non-parametric procedures were used to test and generalize the conclusiveness of the differences in the students’ proficiency levels. The Mann-Whitney U test was used to test for differences in proficiency levels between primary school and farm-based instruction, between sexes, and between the second graders and fourth graders. Friedman’s ANOVA and Wilcoxon’s test were used to test for differences in knowledge level over time, i.e., before the lesson, immediately after it, after 14 days, after one month, and after six months. Statistical significance was set at p < 0.05.

## Results

### Intergroup assessment of the acquired knowledge before the lesson, after its completion, and after 14 days, one month, and six months, taking into account the location of the training

**Fig 1:**
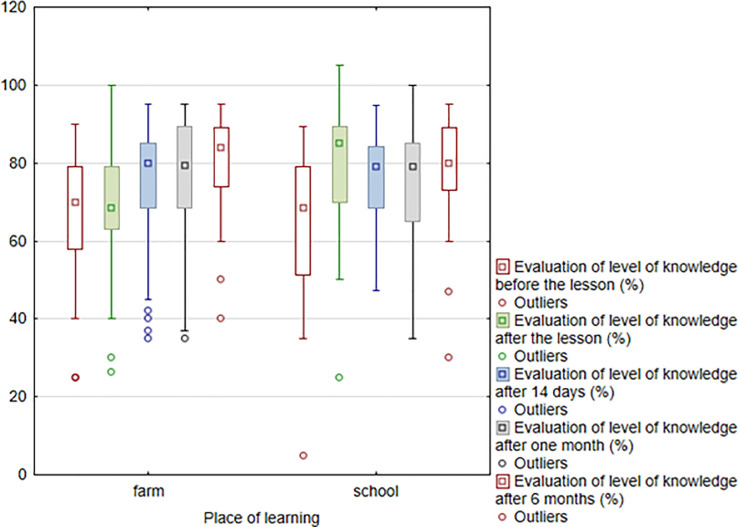
Evaluation of the level of knowledge before, immediately after the lesson, 14 days, one month, and six months after the lesson on the farm and in school (%).

The result of the testing showed that before the lesson, there was no difference in the knowledge between children taught at the farm and children taught in the classroom (p = 0.501). However, when comparing the knowledge of the pupils immediately after the class, the pupils who were taught as usual in the classroom without the presence of animals and change of environment performed significantly better (p = 0.001). At the testing after 14 days, there was no longer a difference (p = 0.848) in pupils’ proficiency levels between the two groups. The same result was observed at the testing after one month (p = 0.760) and after six months (p = 0.649). The summary results can be seen in Fig 1.

Before the lesson, the students taught at the farm averaged 66.9% correct on the test, and after the lesson, they answered 68.9% correctly. After 14 days, they had an average of 74.3% correct answers, so there was an increase in scores. This trend continued as the average score was 76.1% when tested after one month and reached 80.1% after six months.

For the children who attended the lesson in the classroom, the average score before the lesson was 64.9%, whereas the score after the lesson was 79.1%. Thus, the post-lesson score was almost ten percent better than that of children who participated in the lesson at the farm. However, later on, there was a gradual increase in the scores of the children who received the lesson at the farm. There was a decrease in the scores obtained by the children who received the lesson in the classroom after 14 days (76.8%), which continued further. After six months, the average score was 75.3%. Surprisingly, however, this group obtained an average score of 78.6% after one month.

### Intra-group assessment of the knowledge acquired before the lesson, immediately after, and 14 days, one month, and six months after the lesson concerning the lesson location

**Fig 2:**
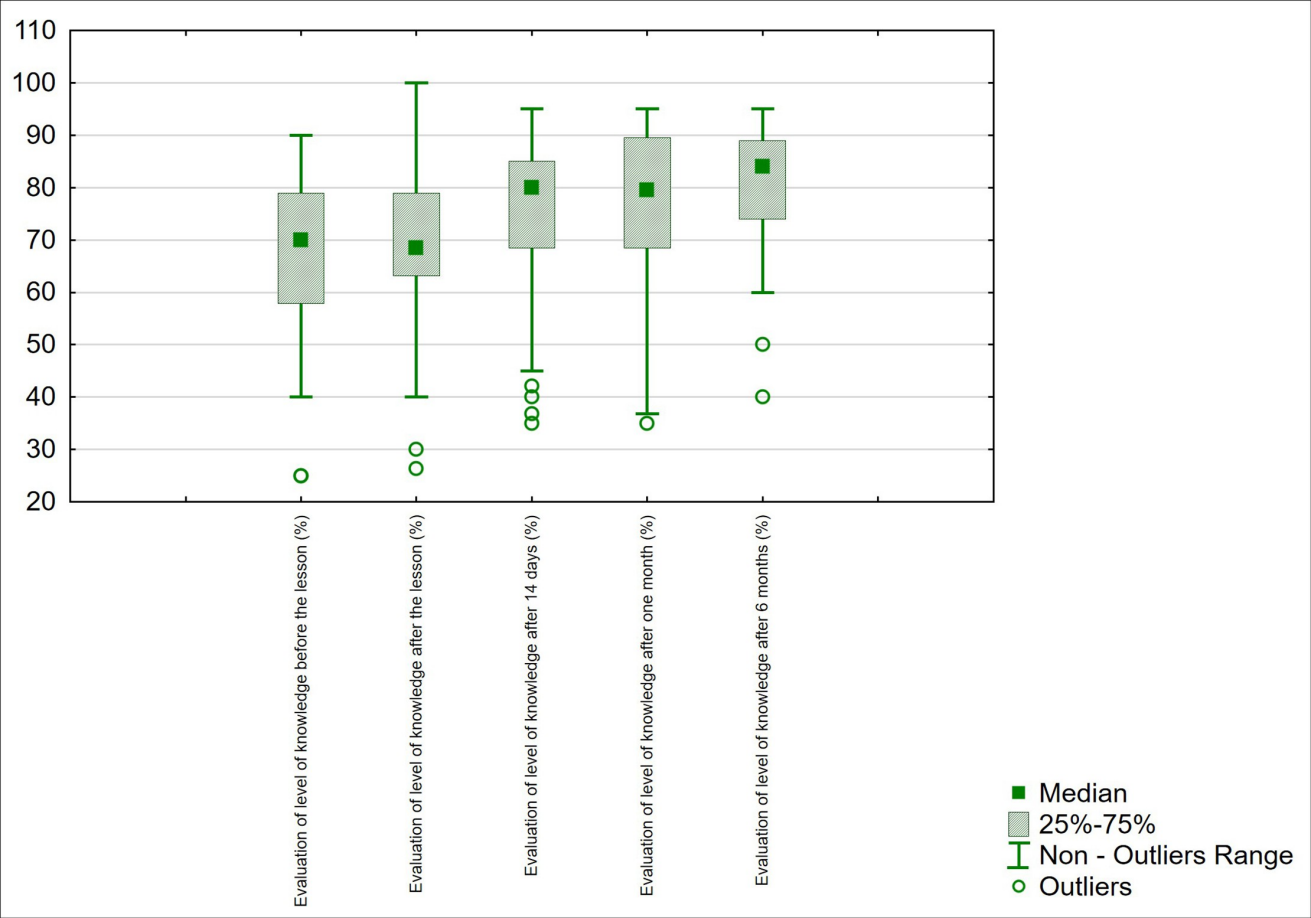
The level and distribution of knowledge of students, who participated in farm education, in time.

Overall, there was a significant difference in the level of pupils’ knowledge over time (p < 0.001) for pupils who attended the lesson at the farm. This difference was not observed when comparing knowledge before and after the lesson (p = 0.438). There was a significant difference when comparing knowledge immediately after the lesson and after 14 days (p = 0.004). The difference between the level of knowledge reported 14 days after the lesson and one month after the lesson was not significant (p = 0.592), as well as when comparing the level of knowledge one month and six months later (p = 0.374). The significance of the differences in testing the level of change in knowledge over time for the students taught at the farm can be found in Table 1. The results obtained for children participating in the lesson at the farm displayed over time can be found in Fig 2.

**Table 1:** The results of testing the levels of knowledge of students who participated in farm education in time.

	Before	After	14 days later	1 month later	6 months later
**After**	p = 0.438	X	X	X	X
**14 days later**	p < 0.001	p < 0.004	X	X	X
**1 month later**	p < 0.001	p < 0.012	p = 0.592	X	X
**6 months later**	p < 0.001	p < 0.001	p = 0.374	p = 0.705	X

**Fig 3:**
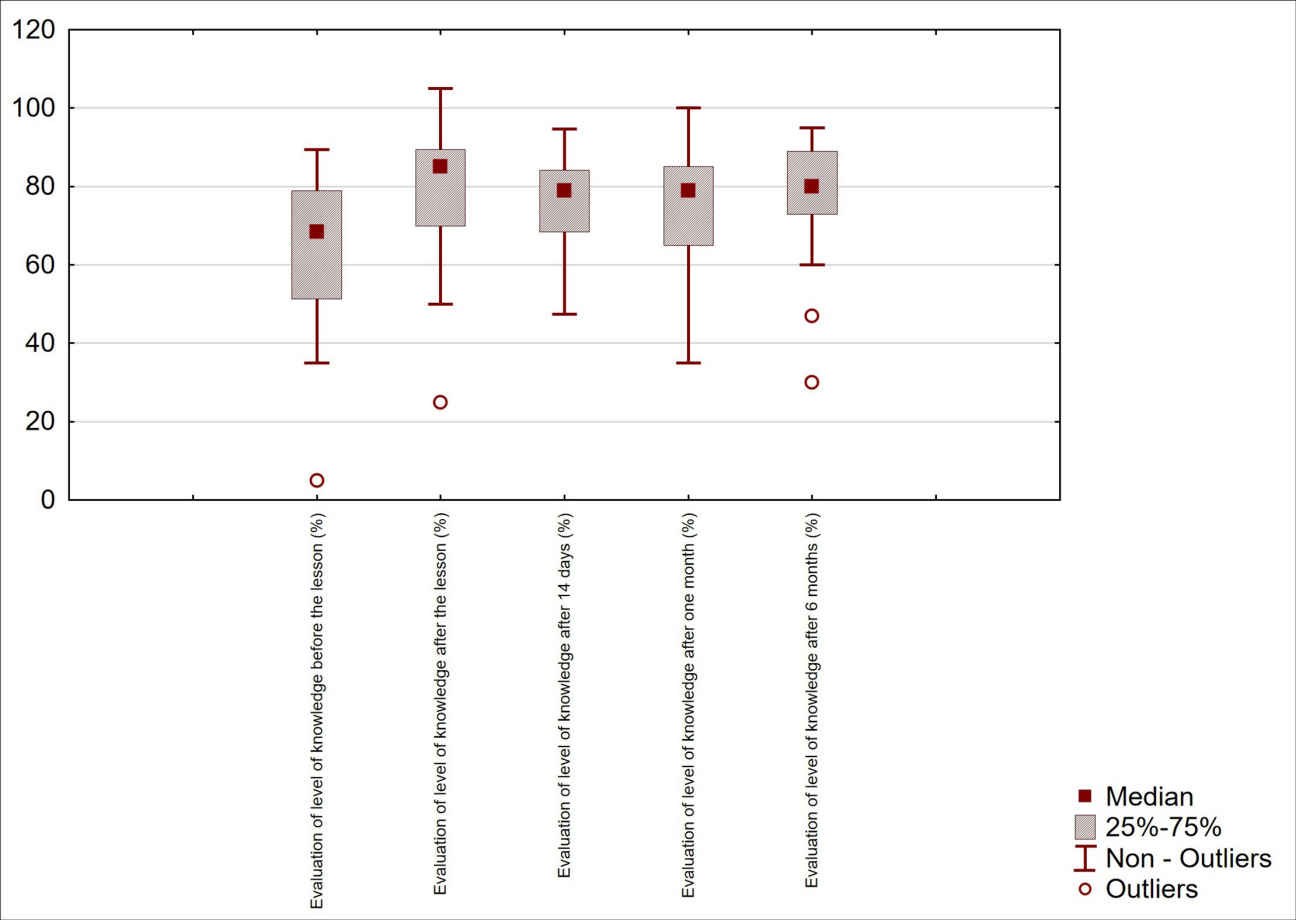
The level and distribution of knowledge of students who participated in school education in time.

Overall, there was no significant difference in students’ proficiency levels over time (p = 0.215) for students who attended the lesson in the classroom. This difference was observed when comparing knowledge before and after the lesson (p < 0.001). No significant difference was observed when comparing knowledge immediately after the lesson and 14 days later (p = 0.193). The difference between the level of knowledge reported 14 days after the lesson and one month after the lesson was not significant (p = 0.294), as well as when comparing the level of knowledge one month and six months later (p = 0.838). The significance of the differences in testing the level of change in knowledge over time for the students taught at the farm can be found in Table 2. The results obtained for the children participating in the lessons at the school displayed over time can be found in Fig 3.

**Table 2:** The results of testing the levels of knowledge of students who participated in school education in time.

	Before	After	14 days later	1 month later	6 months later
**After**	p < 0.001	X	X	X	X
**14 days later**	p = 0.059	p = 0.193	X	X	X
**1 month later**	p < 0.001	p = 0.550	p = 0.294	X	X
**6 months later**	p < 0.001	p = 0.549	p = 0.638	p = 0.838	X

### Assessment of the knowledge acquired before the lesson, immediately after and 14 days, one month, and six months later, taking into account the children’s placement in grades according to age

Before the start of the lesson, there was a difference between the groups (p = 0.012) in favor of the second-grade pupils who answered the questions significantly better before the lesson. In contrast, the fourth-grade pupils answered significantly worse. Second-grade students scored an average of 71.6%, whereas fourth-grade students scored an average of 61.8%.

**Fig 4:**
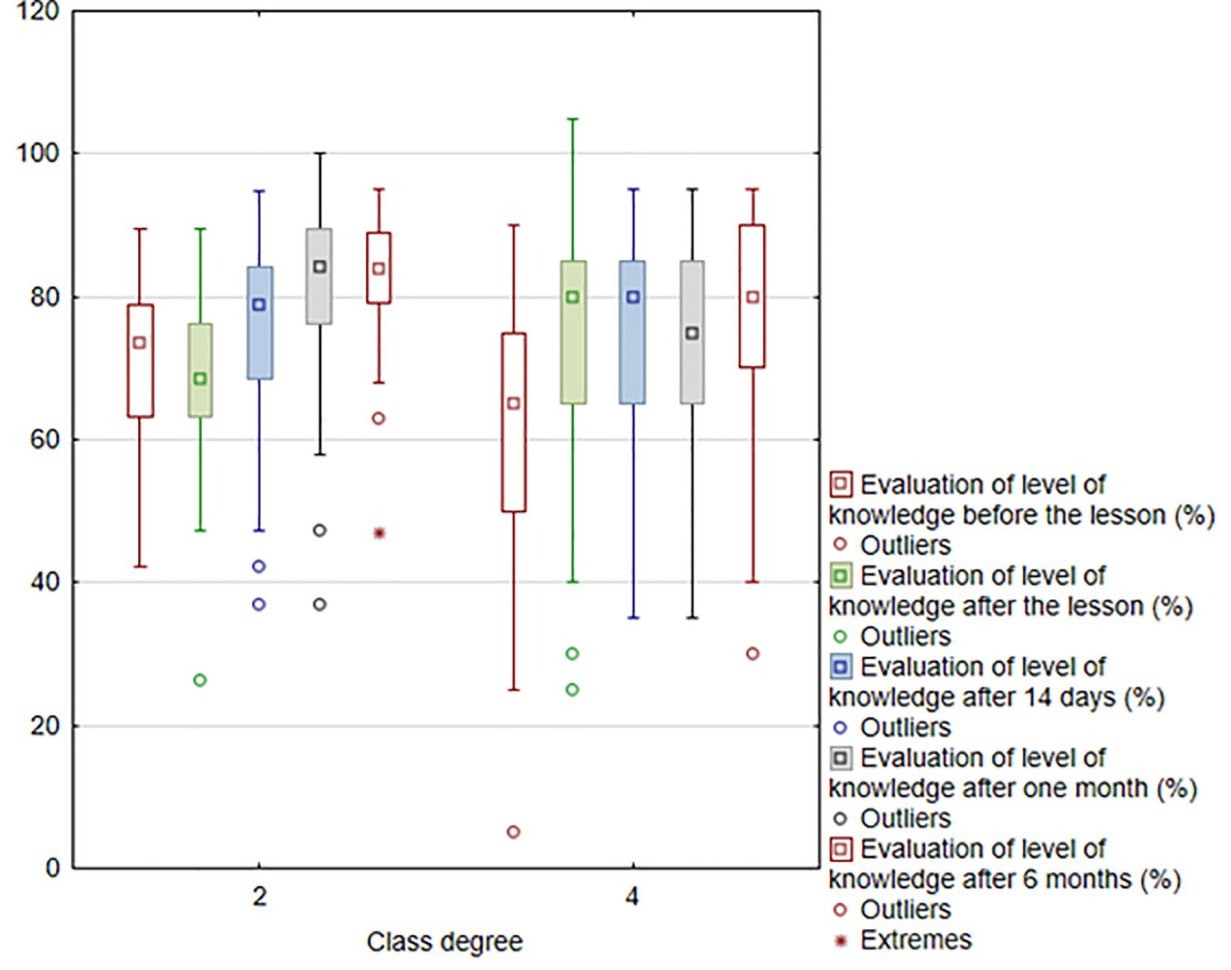
The second and fourth-grade students’ scores at the observed periods, regardless of lesson location.

After the lesson, regardless of the location, second-grade students averaged 70.2%. Later they averaged 76.2%, 79.5%, and 82.2% after 14 days, one month, and six months, respectively. Thus, immediately after the lesson, their scores decreased and then gradually increased over time. On the other hand, the fourth-grade students had 75.9% of the answers correct immediately after the lesson. After 14 days (73.6%) and after a month (73.7%), their scores were slightly lower and, conversely, after six months, slightly higher than immediately after the lesson (76.9%). The scores obtained at the different periods can be seen in Fig 4.

The difference between the knowledge acquired immediately after the lesson by the second (70.2%) and fourth (75.9%) grade students was statistically significant (p = 0.008), but this time in favor of the fourth grade. No significant difference in scores was observed after 14 days (p = 0.802), as well as after one month (p = 0.206) and six months (p = 0.2886).

### Assessment of the knowledge acquired before, immediately after, and 14 days, a month, and six months after the lesson taking into account the children’s placement in grades according to age and the place of the lesson

**Fig 5:**
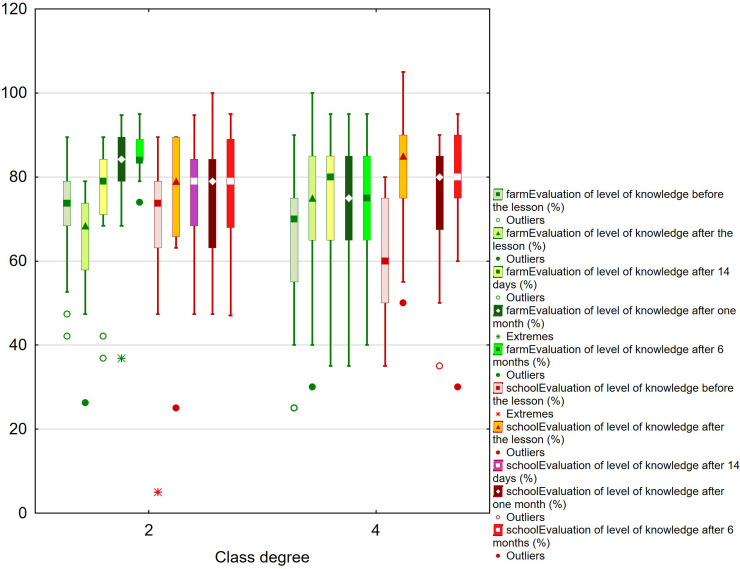
Second and fourth-grade students’ scores at the observed periods concerning the lesson location.

When examining the effect of the environment on second and fourth-grade students, a significant difference in the scores of second-graders immediately after the lesson between the lesson at the farm and the lesson at school was observed (p = 0.003) in favor of students who studied at school. While before the lesson, there was no significant difference in the proficiency level of the second graders (p = 0.958). Furthermore, no difference between the scores obtained by the pupils who studied at the farm and those who studied at school was observed at the assessment after 14 days from the date of the lesson (p = 0.871) or after one month (p = 0.299) or after six months (p = 0.083). Interestingly, this result was already very close to significance, and the students who studied on the farm performed better (Fig 5).

On the other hand, no significant difference in achieved scores was observed for fourth-grade students either immediately after the lesson (p = 0.007) or before it (p = 0.389). It was impossible to test the results 14 days after the lesson because of missing data due to high student absenteeism because of the pandemic and quarantine measures. However, no significant difference was observed either one month after the lesson (p = 0.711) or six months after (p = 0.338) (Fig 5).

### Assessment of the knowledge acquired before, immediately after, and 14 days, a month, and six months after the lesson taking into account the sex of the children and the lesson location

When controlling for the sex of the respondents, no significant difference was observed both before the lesson (p = 0.084) and after it (p = 0.408). Similarly, no difference in knowledge was observed between boys and girls after 14 days (p = 0.259), after one month (p = 0.479) and after six months (p = 0.185).

For second-grade students, their sex did not affect their scores. For both boys and girls, knowledge was comparable before the lesson (p = 0.234), as well as after it (p = 0.487), 14 days after the lesson (p = 0.575), one month (p = 0.121) or six months (p = 0.752).

**Fig 6:**
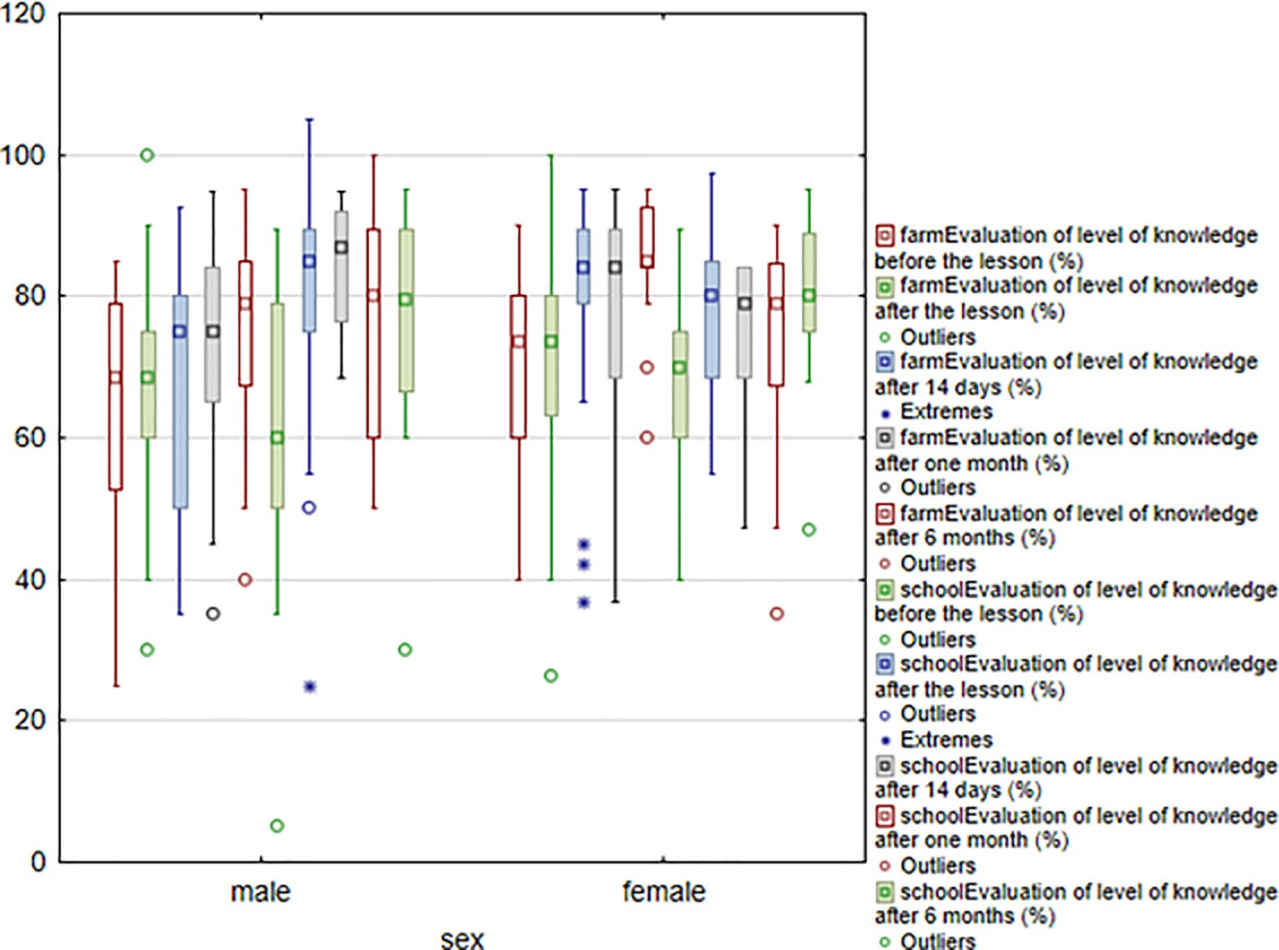
Boys’ and girls’ scores at the observed periods concerning place lesson location.

There was a significant difference in the fourth-grade students’ pre-lesson knowledge (p = 0.017), with boys showing a conclusively higher level of knowledge than girls. However, there no longer proved to be a difference between the students after the lesson (p = 0.757). When students’ knowledge was assessed after 14 days, a significant difference was demonstrated (p = 0.049), with girls demonstrating a higher level of knowledge. After one month, the level of knowledge of boys and girls was the same (p = 0.149). The same was true after six months (p = 0.247) Fig 6.

## Discussion

The effectiveness of the teaching process depends on correctly setting out the objectives and content, as well as the ways to achieve these objectives, i.e., the appropriate choice of the teaching method, organizational form, and material resources available to the teacher [29]. In our study, when knowledge was tested immediately after the lessons, there was a significant difference in the children at school who showed significantly higher knowledge than those who studied at the farm, where this differs from the baseline knowledge was not observed. Later, however, no difference was observed between the groups and their level of knowledge. Children taught at the farm showed the same level of knowledge as those taught at school.

After the lesson, the scores of the students whose lesson occurred at the farm remained almost unchanged compared to their scores before. However, it does not appear that the students at the farm did not acquire the presented knowledge. Instead, the children seemed to be unfocused, distracted, and perhaps full of emotion or excitement after the lesson. Later, their knowledge was comparable to that of their counterparts at school. On the contrary, after 14 days, they scored five percent higher on the assessment test than those who had studied at school. These students may have acquired more information during the lesson at the farm. However, it is also possible that this higher knowledge was due to the children’s interest in the topic being discussed, and they may have pursued it further at home with their parents. An increase in interest in the topic presented could naturally correct the negative trend of children often lacking knowledge regarding the needs of farm animals [26, 27]. However, this has unfortunately not been investigated. After one month and six months, the test scores for both groups were comparable.

The learning process is effective, provided the students enjoy learning and discovering something new. This effect can be achieved through appropriate motivation [9]. Furthermore, it is the increase in motivation and interest that many authors cite in the case of animal-assisted education. Authors Rakotomamonjy et al. [30] state that direct interaction with the object of learning leads to increased interest in the subject being discussed. However, despite these expectations, the students in the experimental group did not score higher than those taught at school. One aspect is what knowledge our students gained, and the other is how the interaction with live animals in their natural environment affected the children’s relationship with the living creatures that were the lesson’s subject. Children today tend to spend most of their time in structured indoor environments [31]. The loss of direct interactions between humans and nature has the potential to increase negative attitudes towards nature (’biophobia’). If biophobia persists into adulthood, it can negatively affect motivation to protect animals and nature [32]. Incorporating teaching with the direct involvement of animals could, in turn, reverse this phenomenon. The presence of animals in educational settings can provide enjoyment and hands-on learning experiences, improve psychological well-being and increase empathy, and promote socio-emotional development [33]. Animals help children recognize their own emotions and the emotions of others. Furthermore, they are a protective factor against significant stress, anxiety, and difficulties in educational programs [34].

Prior to the start of the lesson, second-grade students responded significantly better. These students scored ten percent higher than the fourth graders. The second-grade test can explain that this was about more general knowledge that students may have studied at home with their parents. On the other hand, the knowledge tested in the fourth graders was more detailed. Moreover, the second graders had better scores after the school lesson than those who studied at the farm. Thus, it seems that these students were more distracted by the new environment and the interactive experience, so they could not increase their scores on the presented test when tested immediately after the lesson. However, this does not mean they did not acquire the presented knowledge.

On the contrary, at the testing after six months, the difference in the final scores of the group studying on the farm and the group studying at school approached the level of significance, this time in favor of the students who studied at the farm, who scored better than the students who had six months earlier studied the same material at school. This phenomenon was not observed among the fourth-grade pupils. Their scores were comparable in all conditions.

Another factor influencing the knowledge acquired is that the learning demands should be appropriate to the individual learner’s abilities. In educational practice, this is most often a matter of students’ age appropriateness and respecting the stages of cognitive maturation [20]. If learners understand the material being taught, we can avoid mechanical learning, which is superficial and ineffective [9]. Furthermore, it is the mediation of the experience at the farm and the direct contact with the animals and their young that could influence the learning actually experienced and lived, leaving a more profound mark. It would be interesting to evaluate what knowledge students retained, for example, one year later. Picton et al. [9] report that students remember 10% of what they read, 20% of what they heard, 30% of what they saw, 50% of what they saw and heard, 70% of what they said, and 90% of what they said and did simultaneously. When such interactive learning is included, if properly organized, pupils should be able to see, hear, say and do as part of the learning experience. However, it is always very much up to the teacher how they can implement these different components. Serdyukov [35] states that each lesson should be prepared in such a way that it forms a coherent unit in terms of content, organization, and methodology, which is optimally used in terms of time and pedagogy and should fully engage the pupils. Pupils should experience success, feel good, and be praised [9]. However, children individually differ in their learning styles, vulnerability, coping strategies and stress responsiveness. A task seen by one child as stimulating can be perceived as too stressful by another–and this can shift even with the same individual based on various external or internal factors, such as fatigue, mood, family life etc. Acute elevated stress levels are associated with a rapid decrease in activity in the working memory, planning and response inhibition areas of the brain, while other cognitive functions actually improve, learning included. Still, too much or too little stress is associated with impairment of the learning process. At high stress, children have difficulties with voluntarily controlling their attention, are easily distracted and, due to reduced inhibitory control, are also more inclined to habitual behaviors and indiscipline [36]. However, education in a natural environment could provide several opportunities to brighten up the lessons, lower overall stress levels, allow students to experience a sense of achievement, and give practical meaning to the topics discussed.

Our results show that the knowledge gained from lessons at the farm is comparable to the knowledge gained at school, and therefore there is no need to worry that the lessons are ineffective. On the other hand, Smeds et al. [18] displayed even better results when the learning experience of pupils at a farm even improved compared to the classroom because, as interviews with participants showed, they had an opportunity to build a relationship with a task in its real context and therefore gained deeper and more persistent understanding. When compared to our study, differences between the results of the pupils could be explained by the different curriculum, which might vary between the countries and between the grades, which were also probably slightly different in each study since age of the participants was 9–11 years in the Smeds et al. [18] study and 7–10 years in ours. Even though the topics of lessons were similar in both studies (concentrated on farm and animal related concepts), and in our case based on a national standard educational curriculum, they were possibly more difficult in the Smeds et al. [18] study than in ours. Topics such as ecological and conventional farming, biodiversity, dairy farming, etc. are possibly more difficult and therefore provide better opportunity to reveal the differences between conventional and farm-based education than questions about what the animals eat, products that can be obtained from livestock, body parts of a cow or names of the farm animal offspring.

For future studies, it seems appropriate to include an assessment of parameters such as the level of interest in the topic, student activity during the lesson, and the emotional impact. This, in turn, can affect students’ cooperation, classroom relationships, and relationship with teachers.

It would be interesting to put the results of the knowledge obtained at the farm versus the school in context with the participant’s place of residence. In our case, the students were from a small town. However, it is possible that the results obtained for pupils from a city would have been different due to the minimal opportunity for these children to have contact with farm animals. A study by Burich & Williams [27] highlights the potential of direct interaction with farm animals for increasing children’s well-being by reducing the fear of animals, as well as an increase in compassion for animals. Children who had sporadic contact with farm animals reported more negative experiences, primarily bothered by smells and sounds. The study also reported that children over ten years were interested in discussing the ethical aspects of killing animals for human consumption. In our study, the inclusion of real-life learning environments seems more appropriate for younger children; this would also merit further research. Last but not least, it also seems appropriate to evaluate a long-term learning program that could take place at a farm. In this case, we only evaluated the knowledge gained in one lesson.

Our results significantly contribute to the overall insight into the issue of teaching in an out-of-school environment. The literature on this topic is very limited and there are currently very few articles dealing with this issue. The fact that this method of teaching is effective is of global importance and we hope that it will contribute to the global inclusion of teaching on farms within the study of biology.

### Limits

The primary constraint is the limited number of participants under study. Since this constitutes a pilot study, these initial results represent the starting point for additional research, highlighting the feasibility of incorporating out-of-school education in the traditional teaching process. The results may also be affected by the different numbers of pupils completing the test at each period. Higher absenteeism was caused by the Covid- 19 pandemic. The structure of the lesson can undoubtedly influence the result. However, it was based on the school curriculum and was delivered to the students by an experienced teacher who is also a livestock farmer. The children’s relationship with animals, which was not taken into account in this study, may also significantly influence the results.

The authors are aware of the use of older literary sources as well. Unfortunately, there is very little literature on the topic of involving farm animals in teaching or on the topic of teaching taking place on a farm. This is also the reason for carrying out this study, the aim of which is to contribute to a better understanding of its possible effect on students. The inclusion of animals in teaching seems to be a suitable alternative method of teaching and there is a need to expand the scientific knowledge of this area.

## Conclusion

The scores obtained from the test, conducted immediately after the lesson, showed the lesson taking place in the classroom to be more effective. However, this difference was erased over time, and on subsequent tests, the students who had been taught at the farm were already performing at the same level as the pupils who had studied at school. Interestingly, on closer observation, it was the second-grade students who responded significantly worse immediately after the test at the farm. However, on subsequent tests, they were already performing comparably to the children who had studied at school and even showed a tendency to perform better than the children who had studied at school after six months.

Direct interactions with farm animals offer an increase in children’s well-being in terms of reduced fear of animals, as well as increased compassion for animals. The presence of animals in the educational environment can provide enjoyment and hands-on learning experiences, improve psychological well-being and increase empathy, and even promote socio-emotional development. Animals help children recognize their own emotions as well as the emotions of others. Also, they are often incorporated as a protective factor against significant stress, anxiety, and difficulties in educational programs. Including education with the direct participation of animals could also remove children from the structured indoor environment where they now spend most of their time and reverse this phenomenon.

Since the knowledge acquired by children at the farm is comparable to that gained in a traditional learning environment, it seems that the inclusion of lessons at a farm should not negatively affect the curriculum and may offer many positive influences.

The results of our study show that even if the time allowance for teaching outside the school environment is more demanding, its inclusion in the school education program is ultimately worthwhile because the acquired knowledge is retained for a longer period of time. It also seems that a number of collateral effects are also linked to it, such as arousing interest in the given issue, discussion of the studied material in the home environment, and a shared experience together with the class team. All these additional effects need to be investigated in further studies.

## Supporting information

S1 Data (XLSX)
